# Supplementary material for: Investigation of Bacterial Species and Their Antimicrobial Drug Resistance Profile in Feline Urinary Tract Infection in Thailand
Source: Animals (Basel). 2025 Jul 30;15(15):2235. doi: 10.3390/ani15152235 (PMC12345554; doi:10.3390/ani15152235)
Supplement: Supplementary file 1 [file animals-15-02235-s001.zip › animals-3723799-supplementary.pdf]

Supplementary data

Table S1: Antimicrobial susceptibility percentage of Gram–negative bacteria

**Bacterial species**	**Enterobacteriaceae**					**Non - enterobacteriaceae**		
**Antimicrobial**	*Escherichia coli*	*Proteus mirabilis*	*Klebsiella pneumoniae*	*Enterobacter cloacae complex*	*Enterobacter aerogenes*	*Pseudomonas aeruginosa*	*Acinetobacter spp.*	*Aeromonas spp.*
**Amoxicillin/ clavulanic acids**	54.8	29.4	0.0	0.0	0.0	0.0	0.0	-
**Ampicillin**	32.3	17.6	0.0	0.0	0.0	0.0	0.0	-
Sulfamethoxazole/ **trimethoprim**	48.4	47.1	25.0	50.0	100.0	0.0	33.3	-
**Cephalexin**	61.3	17.6	12.5	0.0	0.0	0.0	0.0	-
**Cephalotin**	38.7	58.8	0.0	0.0	0.0	0.0	0.0	-
**Cefpodoxime**	61.3	64.7	12.5	50.0	100.0	0.0	0.0	-
**Cefovecin**	61.3	76.5	0.0	50.0	100.0	0.0	0.0	-
**Ceftiofur**	64.5	64.7	0.0	50.0	100.0	0.0	0.0	-
**Enrofloxacin**	35.5	41.2	0.0	16.7	100.0	26.7	0.0	-
**Marbofloxacin**	45.2	47.1	0.0	50.0	100.0	53.3	0.0	-
**Pradofloxacin**	42	47.1	0.0	16.7	100.0	0.0	0.0	-
**Doxycycline**	38.7	0.0	12.5	50.0	100.0	0.0	0.0	-
**Tetracycline**	45.2	0.0	12.5	50.0	100.0	0.0	0.0	-
**Gentamicin**	58.0	52.9	62.5	50.0	100.0	66.7	66.7	-
**Amikacin**	93.5	88.2	100.0	66.7	100.0	86.7	0.0	-
**Neomycin**	90.3	94.1	100.0	83.3	100.0	0.0	0.0	-
**Chloramphenicol**	64.5	58.8	0.0	50.0	100.0	0.0	0.0	-
**Nitrofurantoin**	90.3	0.0	87.5	66.7	0.0	0.0	0.0	-
**Imipenem**	96.8	23.5	100.0	83.3	100.0	93.3	33.3	-

- Not tested

Table S2: Antimicrobial susceptibility percentage of Gram–positive bacteria

Bacterial species	*S. felis*	*S. epidermidis*	*S. pseudintemedius*	*S. aureus*	*S. schleiferi*	*E. faecium*	*E. faecalis*	*E. avium*	*E. raffinosus*	*Lactococcus garvieae*	*Corynebacterium urealyticum*	*Streptococcus canis*
**Oxacillin**	100.0	0.0	42.9	100.0	100.0	-	-	-	-	-	-	-
**Cefoxitin screen**	100.0	25.0	-	100.0	100.0	-	-	-	-	-	-	-
**Benzylpenicillin**	45.5	0.0	14.3	100.0	0.0	14.3	100.0	0.0	100.0	-	-	-
**Amoxicillin/ clavulanic acids**	90.9	75.0	42.9	100.0	100.0	14.3	83.3	-	0.0	-	-	-
**Sulfamethoxazole/ trimethoprim**	100.0	100.0	71.4	100.0	100.0	-	-	-	-	-	-	-
**Cefovecin**	100.0	0.0	42.9	100.0	100.0	0.0	0.0	0.0	0.0	-	-	-
**Inducible clindamycin resistance**	90.9	100.0	42.9	100.0	100.0	-	-	-	-	-	-	-
**Enrofloxacin**	100.0	100.0	14.3	100.0	100.0	14.3	16.7	0.0	0.0	-	-	-
**Marbofloxacin**	100.0	100.0	14.3	100.0	0.0	0.0	0.0	0.0	100.0	-	-	-
**Pradofloxacin**	100.0	100.0	14.3	100.0	0.0	-	-	-	-	-	-	-
**Doxycycline**	100.0	100.0	28.6	100.0	100.0	0.0	33.3	100.0	100.0	-	-	-
**Minocycline**	100.0	100.0	100.0	100.0	100.0	-	33.3	0.0	100.0	-	-	-
**Clindamycin**	81.8	0.0	100.0	100.0	100.0	-	-	-	-	-	-	-
**Erythromycin**	90.9	75.0	28.6	100.0	100.0	0.0	0.0	0.0	100.0	-	-	-
**Chloramphenicol**	100.0	100.0	28.6	100.0	100.0	71.4	66.7	0.0	0.0	-	-	-
**Florfenicol**	90.9	100.0	100.0	100.0	100.0	85.7	100.0	0.0	0.0	-	-	-
**Amikacin**	100.0	100.0	100.0	100.0	100.0	-	-	-	-	-	-	-
**Gentamicin**	100.0	75.0	85.7	100.0	100.0	-	-	-	-	-	-	-
**Nitrofurantoin**	100.0	100.0	100.0	100.0	100.0	28.6	100.0	100.0	100.0	-	-	-
**Vancomycin**	-	-	-	-	-	100.0	100.0	100.0	100.0		-	-

- Not tested

Figure S1: Summary of the overall antimicrobial resistance profiles

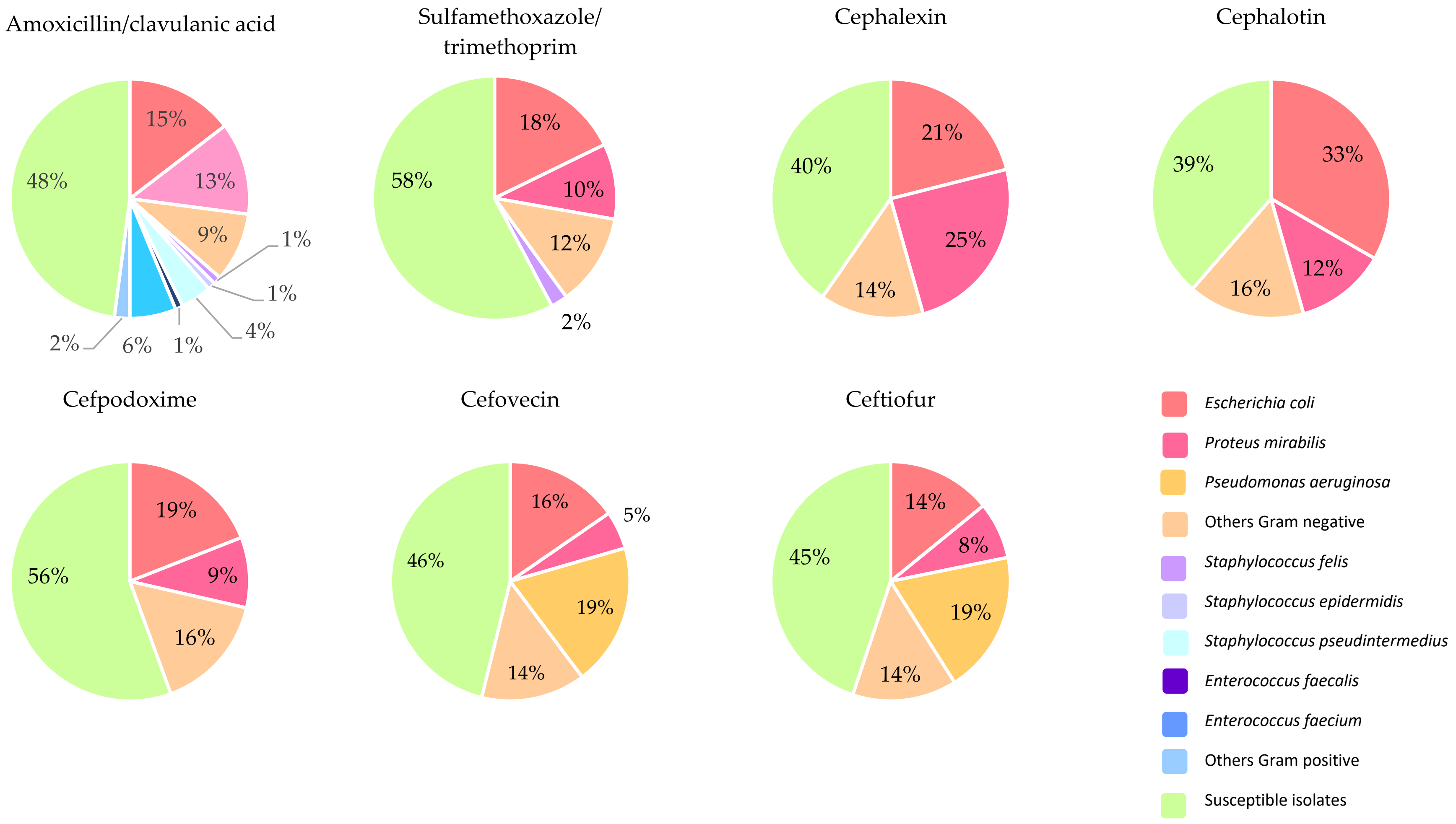

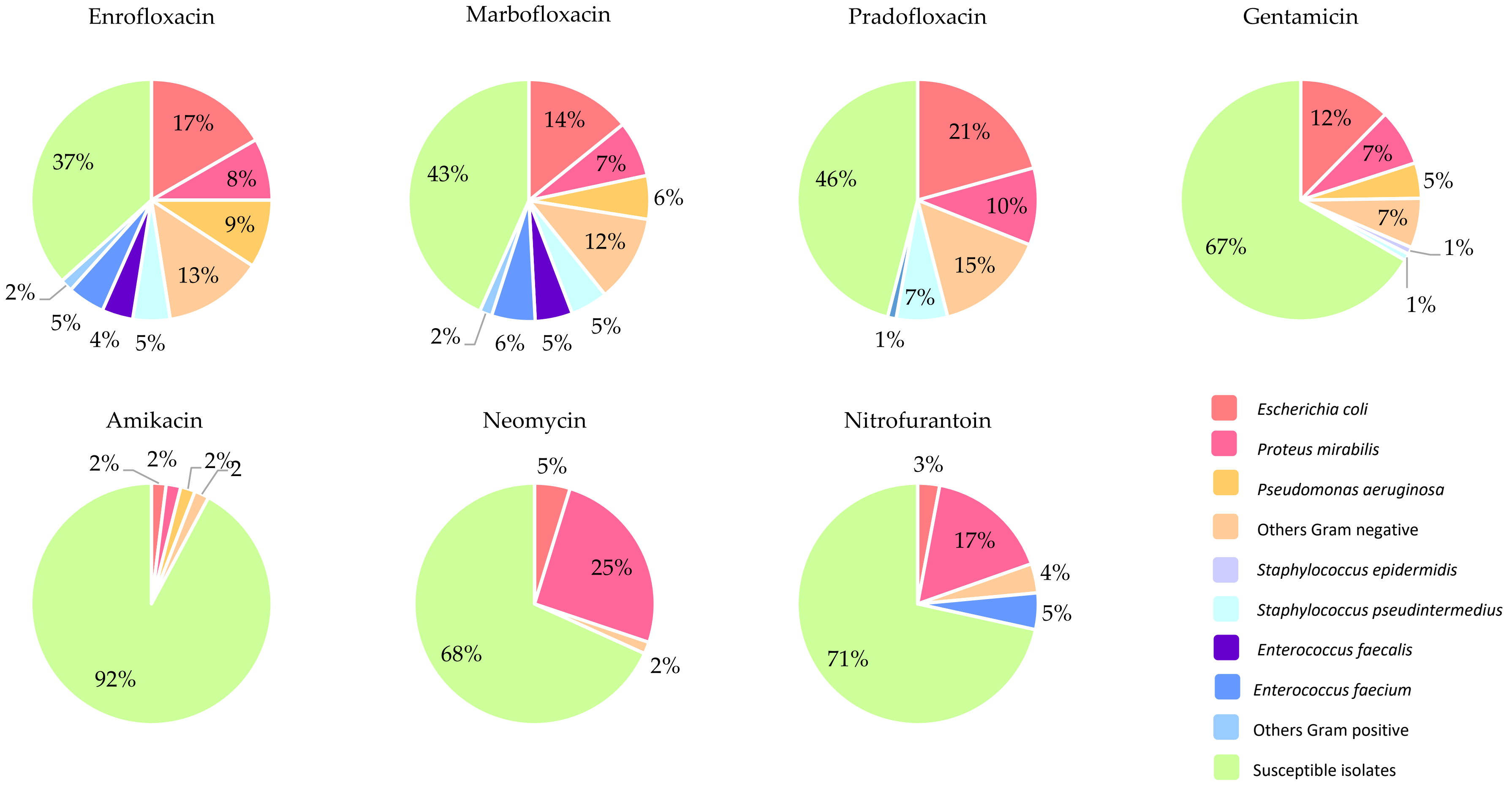
Enrofloxacin
17%
8%
9%
13%
5%
4%
5%
2%
37%
Marbofloxacin
14%
7%
6%
12%
5%
5%
6%
2%
43%
Pradofloxacin
21%
10%
15%
7%
1%
46%
Gentamicin
12%
7%
5%
7%
1%
1%
67%
Amikacin
2%
2%
2%
2
92%
Neomycin
5%
25%
2%
68%
Nitrofurantoin
3%
17%
4%
5%
71%
Escherichia coli
Proteus mirabilis
Pseudomonas aeruginosa
Others Gram negative
Staphylococcus epidermidis
Staphylococcus pseudintermedius
Enterococcus faecalis
Enterococcus faecium
Others Gram positive
Susceptible isolates

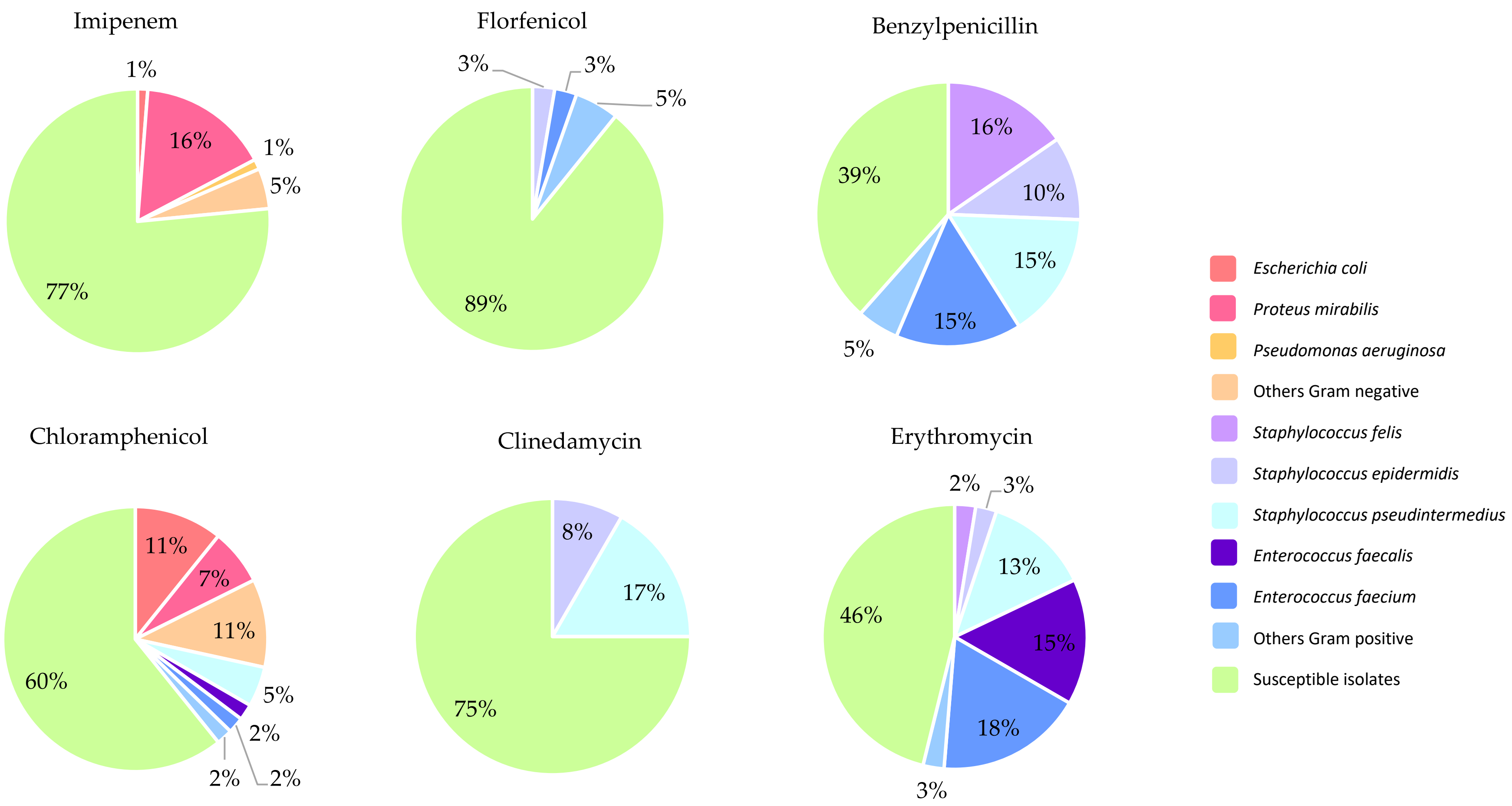
Imipenem
1%
16%
1%
5%
77%
Florfenicol
3%
3%
5%
89%
Benzylpenicillin
16%
10%
39%
15%
15%
5%
Chloramphenicol
11%
7%
11%
60%
5%
2%
2%
2%
Clinedamycin
8%
17%
75%
Erythromycin
2%
3%
13%
46%
15%
18%
3%
Escherichia coli
Proteus mirabilis
Pseudomonas aeruginosa
Others Gram negative
Staphylococcus felis
Staphylococcus epidermidis
Staphylococcus pseudintermedius
Enterococcus faecalis
Enterococcus faecium
Others Gram positive
Susceptible isolates
